# Squelching a Quack

**Published:** 1894-03

**Authors:** 

**Affiliations:** Jerseyville, Ill.


### SELECTIONS.

### Squelching a Quack.

JERSEYVILLE, ILL., Jan. 20, 1894.

*To the Editor:*—The State Board of Health, through Judge O. B. Hamilton, brought suit in this city against one "Dr." Fred Blankner, who was traveling through this region advertising himself and wife as the "champion tooth-pullers of the world," by flaming posters and advertisements in the local papers. This interesting couple also sold electric belts and "yaw soap," which last was recommended as an unfailing cure for sundry and divers diseases. A judgment was recovered against the defendant for $100 and costs, with an order that he stand committed in custody until fine and costs were paid. The wily faker took the train for St. Louis before the jury returned their verdict, and so he is still at large.

The technical charge against him was that of violation of Section 11 of the Medical Practice Act, Chap. 91 R. S. of Illinois, as an itinerant vendor of drugs, without license from the State Board of Health.

The squelching of this quack will doubtless prove beneficial.

Yours truly, MEDICUS.

*Journal American Medical Association.*

